# Predictors of Bacteraemia in Patients with Suspected Community-Acquired Pneumonia

**DOI:** 10.1371/journal.pone.0143817

**Published:** 2015-11-24

**Authors:** Cornelis H. van Werkhoven, Susanne M. Huijts, Douwe F. Postma, Jan Jelrik Oosterheert, Marc J. M. Bonten

**Affiliations:** 1 Julius Center for Health Sciences and Primary Care, University Medical Center Utrecht, Utrecht, The Netherlands; 2 Department of Respiratory Medicine, University Medical Center Utrecht, Utrecht, The Netherlands; 3 Department of Internal Medicine and Infectious Diseases, University Medical Center Utrecht, Utrecht, The Netherlands; 4 Department of Internal Medicine, Diakonessenhuis Utrecht, Utrecht, The Netherlands; 5 Department of Medical Microbiology, University Medical Center Utrecht, Utrecht, The Netherlands; Public Health England, UNITED KINGDOM

## Abstract

**Introduction:**

The diagnostic yield of blood cultures is limited in patients with community-acquired pneumonia (CAP). Yet, positive blood culture results provide important information for antibiotic treatment and for monitoring epidemiologic trends. We investigated the potential of clinical predictors to improve the cost-benefit ratio of obtaining blood cultures.

**Methods:**

Data from two prospective cohort studies of adults with suspected CAP, admitted to non-ICU wards, were combined. Two models were created, one using readily available parameters and one additionally including laboratory parameters.

**Results:**

3,786 patients were included (2,626 (69%) with X-ray confirmed CAP). Blood cultures were obtained from 2,977 (79%) patients (and from 2,107 (80%) with X-ray confirmed CAP). 266 (8.9%) of the patients with a blood culture had bacteraemia. Clinical predictors of bacteraemia were absence of pre-admission antibiotic treatment, pleuritic pain, gastro-intestinal symptoms, tachycardia, tachypnea, hypotension and absence of hypoxia. After including laboratory results in the model, younger age, C-reactive protein, leukocytosis or leukopenia, low thrombocyte count, low sodium level, elevated urea and elevated arterial pH were added, while gastro-intestinal symptoms and hypotension were no longer significant. The area under the receiver operating characteristics curve was 0.66 (95% confidence interval 0.63–0.70) for the first model and 0.76 (95% confidence interval 0.73–0.79) for the second model.

**Conclusion:**

In conclusion, in patients hospitalized with CAP, bacteraemia was moderately predictable using clinical parameters only. We recommend against the use of a risk prediction model for the decision to obtain blood cultures.

## Introduction

Community-acquired pneumonia (CAP) is an infectious disease with a high incidence, especially in elderly or immunocompromized adults, that requires hospitalization in 20–40% of cases.[1,2] Antibiotic treatment is usually empirical, since the causative pathogen is mostly unknown at presentation. Microbiological testing is recommended and positive results may allow pathogen-directed antibiotic treatment.[3–5] Blood cultures are relatively cheap and can be safely obtained. Positive results provide essential information for monitoring longitudinal trends in CAP aetiology and antibiotic susceptibility.[5] However, the advantages for individual patients are less clear. Blood cultures generally suffer from a considerable diagnostic delay, reducing the benefit of streamlining antibiotic treatment.[6] Moreover, reported blood culture positivity in patients hospitalized with CAP has ranged from 5–16%, with growth of common skin contaminants in almost equal proportions of episodes, possibly leading to unjustified changes in antibiotic treatment.[7–12] For these reasons, the recommendation of routinely obtaining blood cultures in all patients with CAP has been questioned. The latest IDSA/ATS guideline recommends to only obtain blood cultures in case of required intensive care unit (ICU) admission, leukopenia, alcohol abuse, severe chronic liver disease, severe obstructive or structural lung disease, asplenia, or if radiologic imaging reveals cavitary infiltrates or pleural effusion.[4] The disadvantage of this approach is that it compromises surveillance of CAP aetiology and antimicrobial resistance. Also, selectively obtaining blood cultures may prohibit pathogen directed therapy in some patients.

To increase efficiency, blood culture drawing could be restricted to patients with a high probability of bacteraemia. Different factors have been associated with bacteraemia in patients with sepsis, including age, previous antibiotic therapy, chills and rigor, vomiting, fever, hypotension, tachycardia, tachypnoea, leukocyte count, thrombocyte count, CRP, creatinine, blood urea nitrogen, and pro-calcitonin.[13–16] In patients hospitalized with X-ray confirmed CAP, absence of prior antibiotic use, chronic liver disease, pleuritic pain, tachycardia, tachypnea, systolic hypotension, temperature, blood urea levels, sodium, and white blood cell count were independent predictors of bacteraemia.[9,17] However, for patient management the domain should not be limited to X-ray confirmed CAP, but include all patients suspected of and treated for CAP and laboratory results (which are generally not available at the time of blood culture taking) should not be included in such models. Therefore, the objective of our study was to identify clinical parameters that predict bacteraemia in patients with clinically suspected CAP admitted to non-ICU wards, and to assess whether a strategy to withhold blood cultures in low-risk patients would be possible without compromising patient management and surveillance of epidemiological trends.

## Materials and Methods

### Study subjects

Data from two multi-centre Dutch cohort studies were combined. The CAP-pilot study was a prospective observational study, conducted in 23 hospitals between January 2008 and April 2009 and the CAP-START study was a cluster-randomized cross-over trial, conducted in 7 hospitals between February 2011 and August 2013. Both the CAP-pilot study (IRB protocol number 07-157/O) and the CAP-START study (IRB protocol number 10-148/C) were approved by the Institutional Review Board of the University Medical Center Utrecht. Patient records were anonymised prior to analysis. The studies' informed consent included permission to use collected data for additional analyses related to the original study. Further details about the design of these studies are described elsewhere.[18,19] From both studies, adult patients of 18 years and above, with clinically suspected CAP, initially admitted to a non-ICU ward, were eligible for this analysis. Demographic data, comorbidities, clinical parameters, laboratory data, X-ray results, and antibiotic use were collected from the medical records by trained research nurses and were anonymously recorded. Blood cultures were taken as part of routine clinical care. Blood cultures yielding coagulase-negative staphylococci and other skin contaminants were not considered to represent bacteraemia. Antibiotic susceptibility testing was performed as part of routine care; these were only available for the CAP-START study. Prior antibiotic use was defined as antibiotic use in the 14 days prior to the current admission.

### Definitions

Clinically suspected CAP was defined as a working diagnosis of CAP for which patients received antibiotic treatment. X-ray confirmed CAP was defined as at least 2 clinical criteria (cough, production of sputum or change in sputum character, temperature >38.0 or <36.1 degrees Celcius, auscultatory findings consistent with pneumonia, leucocyte count > 10*10^9 / L, CRP > 30 mmol/L, and arterial oxygen pressure <8 kPa) and signs of an infiltrate on chest X-ray according to the local radiologist.

### Selection of candidate predictors

Candidate predictors of bacteraemia were selected from the literature and included age, immunocompromised state, chronic liver disease, receipt of pre-hospital antibiotics, gastro-intestinal symptoms, pleuritic pain, chills, confusion, hypotension (systolic blood pressure below 90 mmHg or diastolic blood pressure below 60 mmHg), tachycardia (heart rate above 125 / min), tachypnea (respiratory rate above 30 / min), hypoxia (oxygen saturation below 90% without oxygenation), C-reactive protein, body temperature, leukocyte count, thrombocyte count, sodium, urea, glucose, arterial pH, and presence of an infiltrate on chest X-ray.[9,13–17]

### Analysis

Continuous predictors were assessed for linearity with the outcome by visual inspection of Lowess curves. Variables with no linear association were entered in the model using a piecewise linear function, which generates separate regression lines for the variable below and above a specified break point.[20] Missing data were handled using Multivariate Imputation by Chained Equations, [21] except for confusion, gastro-intestinal symptoms, and chills. These were assumed to be absent if not documented in the medical records. Fifty imputed datasets were created.

Prediction models were derived using multivariable logistic regression with bacteraemia as the outcome variable. Starting from the model with all candidate predictors, stepwise backward elimination was used to identify independent predictors with a p-value below 0.1 using the likelihood ratio test. Two models were created, one only including candidate predictors that are available at the time of blood culture collection, and one also including laboratory and radiology results. The models were internally validated using 200 bootstrap samples. Performance of the models was assessed using the Area Under the Curve (AUC) of the Receiver Operator Characteristic, sensitivity, specificity, and positive and negative predictive values at different cut-off values of the predicted risk. As a sensitivity analysis, the AUC was also determined in patients with X-ray confirmed CAP. Performance of previously published models [9,17] was assessed similarly.

Using the model with readily available data, the optimal cut-off value was chosen such that discrimination was largest. For patients with a predicted risk below the cut-off value, lost opportunities by omitting the blood cultures were described. This was defined as (1) missing *S*. *pneumoniae* bacteraemia together with a negative pneumococcal urinary antigen test (PUAT); (2) missing antibiotic resistant pathogens; (3) missing pathogens that are intrinsically resistant to usual empiric antibiotic treatment of CAP.

Patients in whom blood cultures had not been obtained were excluded from the analysis of predictors of bacteraemia. To assess which factors were associated with blood culture drawing, we used logistic regression, using the same candidate predictors. All analyses were performed in R version 3.0.2.[22]

## Results

Together, 3,786 patients were initially hospitalized to a non-ICU ward with clinically suspected CAP: 1,503 in the CAP-pilot study and 2,283 in the CAP-START study. Baseline characteristics were comparable between the studies, except for being immunocompromised, which was more frequent in the CAP-START study (Table 1). CAP was confirmed by X-ray in 2,626 patients (69%). Blood cultures were drawn in 2,977 patients (79%), of whom 2,107 (71%) had X-ray confirmed CAP. Bacteraemia was present in 266 (8.9%) of all patients with a blood culture obtained, mostly with *Streptococcus pneumoniae* (Table 2), and an additional 91 (3.1%) patients had positive blood cultures with skin contaminants.

**Table 1:** Baseline characteristics of the study populations.

	CAP-pilot (N = 1,503)	CAP-START (N = 2,283)	Combined (N = 3,786)	Missing data (%)
Age	68.28 (15.09)	67.48 (15.71)	67.79 (15.47)	0%
Male gender	913 (60.8%)	1,317 (57.7%)	2,230 (58.9%)	0%
Immunocompromised	197 (13.1%)	533 (23.3%)	730 (19.3%)	0%
Previous antibiotics	495 (33.5%)	749 (33.6%)	1,244 (33.6%)	2.1%
Temperature (°C)	38.21 (1.15)	38.11 (1.07)	38.15 (1.10)	2.6%
Chills	197 (13.1%)	452 (19.8%)	649 (17.1%)	0%
Confusion	161 (10.7%)	204 (8.9%)	365 (9.6%)	0%
Gastrointestinal symptoms	131 (8.7%)	304 (13.3%)	435 (11.5%)	0%
Heart rate > = 125 / min	166 (11.8%)	287 (12.9%)	453 (12.5%)	4%
Respiratory rate > = 30 / min	136 (22.0%)	255 (17.4%)	391 (18.7%)	44.9%
Hypotension*	219 (15.3%)	311 (13.9%)	530 (14.4%)	2.8%
Hypoxia^	255 (18.5%)	369 (19.4%)	624 (19.0%)	13.2%
Leukocytes (10^9^/L) ^#^	13 (9.6–17.3)	13.1 (9.4–17.8)	13.1 (9.5–17.5)	0.3%
Thrombocytes (10^9^/L) ^#^	NA†	250 (191–326)	250 (191–326)	41.2%
CRP (mg/L) ^#^	103 (38–219)	118 (54–222)	114 (46–221)	1.5%
Sodium (mmol/L)	136.31 (7.33)	136.43 (4.27)	136.38 (5.68)	0.8%
Urea (mmol/L)	6.9 (5–9.6)	6.6 (4.7–9.3)	6.7 (4.8–9.4)	4%
Glucose (mmol/L) ^#^	7.1 (6.1–8.5)	7.1 (6.2–8.5)	7.1 (6.1–8.5)	6.8%
Arterial pH	7.43 (0.10)	7.45 (0.06)	7.44 (0.08)	18.2%
Presence of infiltrate	1,041 (69.3%)	1,585 (69.4%)	2,626 (69.4%)	0%
Blood cultures taken	1,240 (82.5%)	1,737 (76.1%)	2,977 (78.6%)	0%

Data given as mean (SD) or N (%), unless otherwise indicated.

^#^ median (inter-quartile range).

* defined as systolic blood pressure < 90 mmHg or diastolic blood pressure < 60 mmHg.

^^^ Hypoxia was defined as oxygen saturation below 90% without oxygenation.

^†^ Thrombocyte count was not recorded in the CAP-pilot study. NA: not available.

**Table 2:** pathogens isolated from blood cultures and numbers classified as low or high risk by the model without laboratory results (using 60% sensitivity target).

Pathogens	Total *	Risk < 0.09	Risk > = 0.09	% in low risk group
*Streptococcus pneumoniae*	167	62	105	37%
Other Streptococcus species	19	6	13	32%
*Staphylococcus aureus*	15	8	7	53%
Other gram-positives^1^	4	2	2	50%
Enterobacteriaceae^2^	47	21	26	45%
*Haemophilus influenzae*	7	2	5	29%
*Pseudomonas aeruginosa*	7	5	2	71%
Other gram-negatives^3^	6	3	3	50%

^1)^ *Enterococcus faecalis* (3x), unspecified gram-positive coccus.

^2)^ *Citrobacter diversus*, *Enterobacter cloacae*, *Escherichia coli* (31x), *Klebsiella oxytoca* (5x), *Klebsiella pneumoniae* (4x), *Morganella morganii*, *Proteus mirabilis* (3x), Salmonella enteritidis.

^3)^ Acinetobacter species, *Capnocytophaga canimorsus*, *Fusobacterium necrophorum*, Moraxella species, *Porphyromonas asaccharolytica*, unspecified gram-negative rod.

* Six patients had two different pathogens, making the total number of patients with a pathogen 266: *E*. *coli* + *K*. *oxytoca*, *E*. *coli* + *K*. *pneumoniae* (2x), *E*. *cloacae* + *P*. *mirabilis*, *P*. *aeruginosa* + *P*. *mirabilis*, and *P*. *aeruginosa* + *S*. *aureus*.

### Prediction of bacteraemia

A piecewise linear function was needed for temperature (breakpoint at 38°C), leukocyte count (breakpoint at 9.5 * 10^9^/L), glucose (breakpoint at 6.5 mmol/L) and urea (breakpoint at 7 mmol/L). Additionally, leukocyte count and urea level were right-truncated at 40 * 10^9^/L and 15 mmol/L, respectively, since higher values were rare and not discriminative in univariate analysis.

In the first model, only using data available at the time of blood culture collection, independent predictors of bacteraemia were: absence of pre-admission antibiotic treatment, pleuritic pain, gastro-intestinal symptoms, tachycardia, tachypnea, hypotension and absence of hypoxia (Table 3). Discrimination of the model was poor, with an AUC of 0.66 (95% CI 0.63–0.70) both in the full cohort and in the subset of patients with X-ray confirmed CAP (S1 and S2 Figs). In internal validation, optimism of the model was 1.9%, yielding a bias-corrected AUC of 0.64 (95% CI 0.59–0.69). After adding laboratory and radiology results to the candidate predictors, gastro-intestinal symptoms and hypotension were no longer statistically significant, while the following variables were added to the model: younger age, C-reactive protein, leukocyte count, thrombocyte count, sodium level, urea level and arterial pH (Table 4). Discrimination of this model was moderate, with an AUC of 0.76 (95% CI 0.73–0.79) in the total population and 0.76 (95% CI 0.73–0.80) in patients with X-ray confirmed CAP (S3 and S4 Figs). In internal validation, optimism of the model was 2.1%, yielding a bias-corrected AUC of 0.74 (95% CI 0.70–0.78).

**Table 3:** Model without laboratory results of predictors independently associated with bacteraemia.

	Beta	OR	95% CI
(Intercept)	-2.533		
Previous antibiotics within 14 days before admission	-0.786	0.456	(0.326 to 0.637)
Pleuritic pain	0.565	1.760	(1.279 to 2.421)
Gastro-intestinal symptoms	0.547	1.727	(1.237 to 2.413)
Heart rate > = 125/min	0.564	1.757	(1.269 to 2.433)
Respiratory rate > = 30/min	0.421	1.524	(1.085 to 2.139)
Hypotension §	0.486	1.627	(1.179 to 2.244)
Hypoxia ‡	-0.348	0.706	(0.484 to 1.029)

OR: Odds ratio. CI: confidence interval.

^§^ Hypotension was defined as diastolic blood pressure < 60 mmHg or systolic blood pressure <90 mmHg.

^‡^ Hypoxia was defined as oxygen saturation below 90% without oxygenation.

**Table 4:** Model including laboratory results of predictors independently associated with bacteraemia.

	Beta	OR	95% CI
(Intercept)	-13.957		
(+ intercept if leukocytes > 9.5) †	-0.884		
(+ intercept if urea > 7) †	1.754		
Age *	-0.009	0.991	(0.981 to 1.001)
Previous antibiotics within 14 days before admission	-0.698	0.498	(0.352 to 0.703)
Pleuritic pain	0.596	1.815	(1.281 to 2.570)
Heart rate > = 125/min	0.536	1.709	(1.211 to 2.413)
Respiratory rate > = 30/min	0.406	1.501	(1.056 to 2.133)
Hypoxia ‡	-0.384	0.681	(0.459 to 1.012)
C-reactive protein (mg/L) $	0.024	1.024	(1.013 to 1.034)
Leukocytes (values < = 9.5*10^9/L) * #	-0.052	0.949	(0.869 to 1.036)
Leukocytes (values > 9.5*10^9/L) * #	0.041	1.041	(1.021 to 1.062)
Trombocytes (10^9/L) $	-0.017	0.983	(0.968 to 0.998)
Sodium (mmol/L) *	-0.027	0.974	(0.944 to 1.005)
Urea (values < = 7 mmol/L) * ^	0.329	1.389	(1.195 to 1.615)
Urea (values > 7 mmol/L) * ^	0.078	1.081	(1.024 to 1.142)
Arterial pH @	0.186	1.204	(0.984 to 1.474)

OR: Odds ratio. CI: confidence interval.

* per unit increase.

^$^ per 10 units increase.

^@^ per 0.1 units increase.

^#^ right truncated at a value of 40*10^9/L.

^^^ right truncated at a value of 15 mmol/L.

^†^ Leukocyte count and urea are modelled using piecewise linear transformation, therefore, an additional intercept has to be added to the regression line for patients with a value above the break point.

^‡^ Hypoxia was defined as oxygen saturation below 90% without oxygenation.

**Fig 1:**
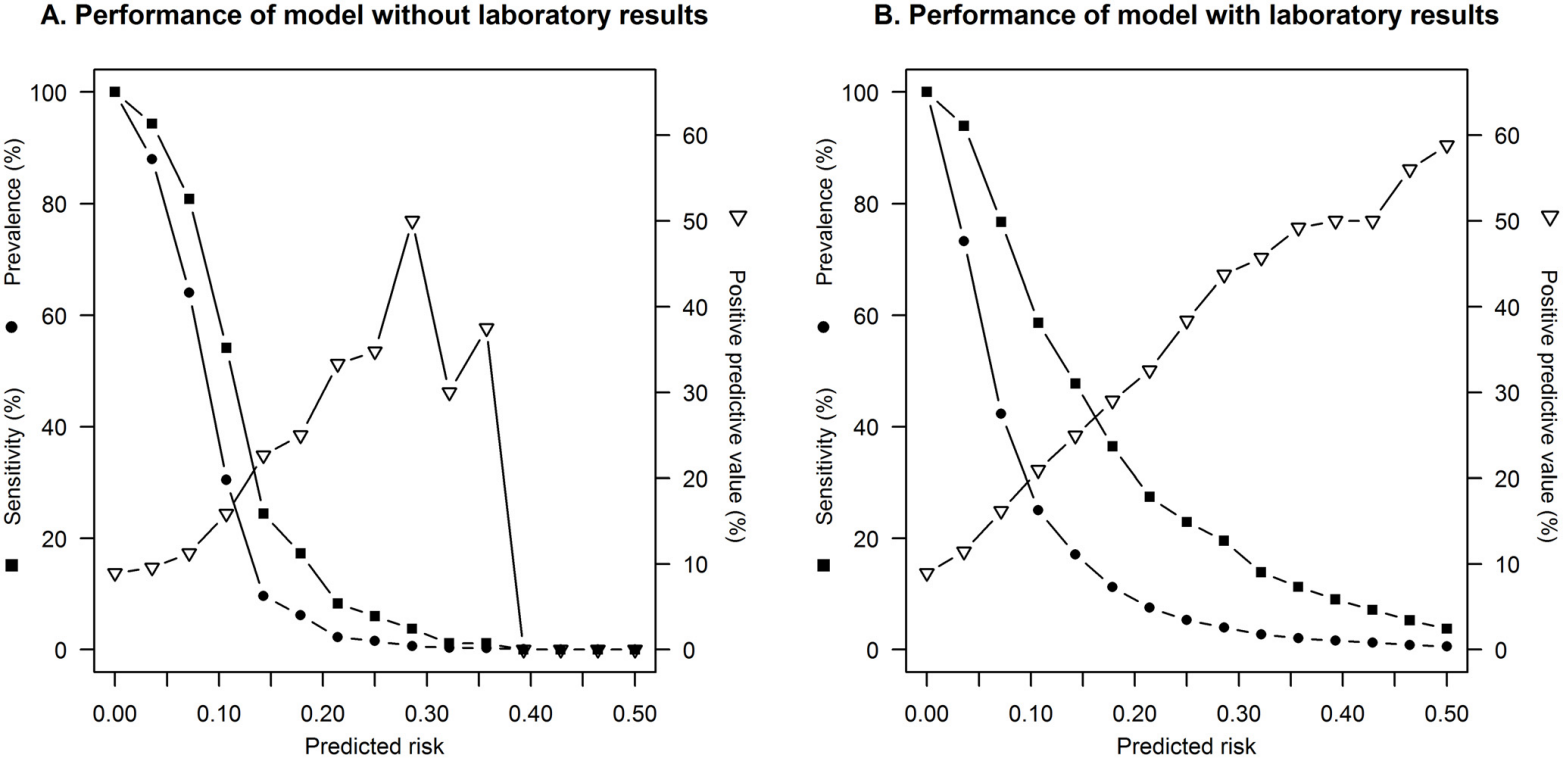
Performance of the model without (A) and with (B) laboratory results. For different cut-off values of the predicted risk, the prevalence of having an equal or higher risk, the sensitivity of this cut-off value, and the positive predictive value are plotted.

Performance of these models is given in Fig 1. The maximum discrimination of the model without laboratory results was reached using a predicted risk of 9% as the cut-off value, which yielded a sensitivity of 60%. 35% of the patients had a predicted risk above 9%. For the model including laboratory results, maximum discrimination was reached using a risk cut-off of 8%, which yielded a sensitivity of 73%. 37% of the patients had a predicted risk above 8%.

Of two previously published models not using laboratory results, the model by Falguera et al.[9] had an AUC of 0.64 (95% CI 0.61–0.68), and the model by Metersky et al.[17] had an AUC of 0.62 (95% CI 0.59–0.65) in our data. The model by Metersky et al.[17] that included laboratory results had an AUC of 0.69 (95% CI 0.66–0.73) in our data.

### Implications for patient management

We, hypothetically, applied the model without laboratory results to the current patient population and assumed that blood cultures were omitted in patients with a predicted risk below 9% (low risk group, i.e. 65% of the total population). In that scenario, 109 (40%) of all pathogens would have been missed (Table 2). Among the 62 episodes of *S*. *pneumoniae* bacteraemia in the low-risk group, 30 (48%) were PUAT positive which would have allowed streamlining of therapy. Yet, 24 (39%) were PUAT negative (sensitivity 56%) and in 8 (13%) episodes PUAT was not performed. Comparable PUAT results were obtained in the 167 *S*. *pneumoniae* bacteraemia cases in the high-risk group: 45% were positive, 46% negative, and 10% not tested.

Of 157 patients with bacteraemia in the CAP-START study, data on antibiotic susceptibility were available for 126 (80%) pathogens, all in unique patients (47 patients in the low risk group and 79 in the high-risk group). Resistance to penicillin / amoxicillin, the recommended antibiotic treatment in this patient population according to Dutch guidelines, [5] was observed in ten patients in the low-risk group (4 *Escherichia coli*, 1 Haemophilus influenza, 1 *Klebsiella oxytoca*, 3 *Staphylococcus aureus*, and 1 Moraxella species) and ten in the high-risk group (3 *E*. *coli*, 2 *H*. *influenza*, 1 *K*. *oxytoca*, 1 *K*. *pneumoniae*, 1 *Morganella morganii*, and 2 *S*. *aureus*). Resistance to amoxicillin-clavulanic acid or cephalosporins was found in only 1 patient with *M*. *morganii* bacteraemia in the high-risk group.

Of seven patients with *Pseudomonas aeruginosa* isolated from the blood culture, five were in the low risk group, of which two were empirically treated with ciprofloxacin. In one other patient *P*. *aeruginosa* was isolated from sputum. The remaining two were empirically treated with amoxicillin-clavulanic acid and sputum culture was not performed in one and negative in the other patient.

### Predictors of obtaining blood cultures

Blood cultures were not obtained from 809 (21%) patients. Presence of hypothermia or fever had a strong association with obtaining blood cultures, while younger age, confusion, presence of chills, tachypnea, elevated CRP level, leukopenia or leucocytosis, thrombocytopenia or thrombocytosis, and elevated blood glucose level were all weak predictors of obtaining blood cultures (S1 Table).

## Discussion

In patients hospitalized with clinically suspected CAP, seven readily available parameters and six parameters available after laboratory examination were independent predictors of bacteraemia. The model without laboratory results was marginally predictive with a bias-adjusted AUC of 64%, while the model with laboratory results had a bias-adjusted AUC of 74%.

The first model had similar discriminative power as two previously published models.[9,17] Apparently, accurate prediction of bacteraemia is not feasible without using laboratory results. The slightly better performance of our second model compared to a previously published model may result from using continuous variables and piecewise linear transformations for the laboratory results.

It has been argued that blood cultures should not be obtained in all CAP patients, but only in patients at high risk of bacteraemia.[7,9–11] However, such a policy would have led to several missed opportunities for optimizing antibiotic treatment in our population. By far the most frequently identified pathogen by blood culture is *S*. *pneumoniae*. Identification of this pathogen provides the opportunity to deescalate antibiotic treatment to penicillin or amoxicillin. This could also be achieved with urinary antigen testing, yet, the sensitivity of this test in bacteraemic patients appeared to be only 52% in our cohort. This is low compared to previously reported sensitivities that range from 65% to 92%.[5] Consequently, omitting blood cultures decreases the proportion of patients in which physicians feel comfortable to switch to narrow-spectrum antibiotics. Antibiotic resistance was present in only a few bacteraemia isolates and there was no obvious difference between patients at low or high risk of bacteraemia. Although a relatively low proportion of CAP patients had resistant pathogens isolated from blood, these will obviously have important consequences for antibiotic treatment, in particular in the few cases of *P*. *aeruginosa* bacteraemia that were not covered by empirical treatment and in whom *P*. *aeruginosa* was not found in sputum cultures.

Pathogens obtained from blood cultures are also important for monitoring epidemiological trends in CAP aetiology and antibiotic resistance. Use of a model to omit blood cultures in low-risk patients will reduce the number of pathogens identified, which may be acceptable. For the model with readily available parameters, the optimal discrimination was reached at a predicted risk of 9%, yielding a sensitivity of 60%. Compared to the current situation, blood cultures could be omitted in 65% of patients when using this cut-off value, of which 40% is due to setting the sensitivity target and 25% is due to selection of high-risk patients by the model. More importantly, use of the model may also affect the distribution of detected pathogens or resistance patterns, e.g. if the model is more sensitive to certain pathogens. In our study, there were no clear differences in distributions of pathogens or in proportions of resistance between the low and high risk patients, but the numbers are too small to draw firm conclusions.

Models including laboratory results predicted bacteraemia better. Yet, in patients presenting with CAP, blood cultures are usually collected at the same time as blood samples for laboratory results, and changing this practice to allow additional sampling for blood cultures in selected patients seems unrealistic.

Models predicting the risk of bacteraemia could still be beneficial for research purposes. For example in trials with a minimum required proportion of bacteraemic patients, inclusion could be based on the risk of bacteraemia. Also, adjustment for risk of bacteraemia in observational studies can be achieved using the model.

In contrast to previous studies, our domain consisted of patients with clinically suspected CAP. Since these are the patients typically managed according to CAP guidelines, our study domain ensures generalizability of our findings to clinical practice. For example, for patients that turn out to have a different source of infection, obtaining blood cultures may still be beneficial, even though the clinical suspicion of CAP is not confirmed. On the other hand, the inclusion of patients with a different source of infection or non-infectious disease may have increased heterogeneity in clinical presentation, thereby hampering accurate prediction. For this reason, we performed a sensitivity analysis in patients with X-ray confirmed CAP, which yielded the same AUCs.

Some limitations of the study need to be addressed. First, blood cultures were not obtained in 21% of patients and factors predicting bacteraemia and obtaining blood cultures partly overlapped, which may have attributed to the moderate discrimination. In particular, abnormal body temperature had a strong association with obtaining blood cultures, but was not a predictor of bacteraemia; the latter may have resulted from selection bias due to the presence of fever or hypothermia. Second, although most candidate predictors were selected from previous publications, we used urea instead of creatinine, because creatinine was not available in our study data. Urea is also used in the CURB-65 score, widely used to predict 30-day mortality in CAP, [23] and appears to be a strong predictor of bacteraemia. Procalcitonin is a known predictor of bacteraemia in sepsis, [24] but is currently not obtained in routine clinical practice and is, therefore, not useful for clinical prediction. Prior antibiotic use was associated with a reduction of the yield of blood cultures by 50%. Unfortunately we had no data on the timing of in-hospital antibiotic use and blood culture collection. Obviously, collection of blood cultures before administration of antibiotic therapy should always be attempted. Third, data on respiratory rate, thrombocyte count, arterial pH, and hypoxia were frequently missing, which may have compromised our analysis. However, consistent parameter estimates were derived from individual imputed datasets (data not shown). Fourth, the collection of data from the medical chart may not fully represent what would have been recorded by the clinician if he would need to complete the risk score, possibly affecting generalizability of the results. Fifth, combining two study cohorts may compromise the study if different in- and exclusion criteria are used. In the CAP-pilot study, patients with known bronchial obstruction, pulmonary malignancies or metastases, AIDS, a history of *Pneumocystis jirovecii* pneumonia, and active tuberculosis were excluded. Although each of these conditions is relatively rare, the proportion of immunocompromised patients in this study was lower compared to the CAP-START study. Other baseline characteristics and the proportion of bacteraemic patients were comparable. Last, we did not split data into a derivation and validation cohort. Instead we used internal validation to assess model optimism, which turned out to be small. The use of a piecewise linear function for urea and leukocyte counts, with a break point based on our own data, might reduce generalizability of the model including laboratory data. If so, this would only further support our finding that prediction models of bacteraemia in CAP have moderate discrimination.

It could still make sense to restrict blood cultures to patients that are likely to benefit from a positive result, such as immunocompromised patients, or that are likely to inform epidemiological trends. As this requires interpretation of the relative importance of positive blood cultures, this should be evaluated in a prospective study.

In conclusion, clinical parameters can only moderately predict the presence of bacteraemia. Given the limited added value of the model, the relatively low costs and non-invasiveness of the blood culture, and the potential benefits for patient management and surveillance of epidemiological trends, we recommend against the use of a risk prediction model for the decision to obtain blood cultures.

## Supporting Information

S1 Dataset Spreadsheet containing the data used in this study. (CSV)

S1 Fig ROC-curve of the model without laboratory results in clinically suspected CAP patients. Abbreviations: ROC: receiver operating characteristic; AUC: area under the curve.
(TIFF)

S2 Fig ROC-curve of the model without laboratory results in X-ray confirmed CAP patients. Abbreviations: ROC: receiver operating characteristic; AUC: area under the curve. X-ray confirmed CAP is defined as presence of at least 2 clinical criteria and signs of an infiltrate on chest X-ray.
(TIFF)

S3 Fig ROC-curve of the model with laboratory results in clinically suspected CAP patients. Abbreviations: ROC: receiver operating characteristic; AUC: area under the curve.
(TIFF)

S4 Fig ROC-curve of the model with laboratory results in X-ray confirmed CAP patients. Abbreviations: ROC: receiver operating characteristic; AUC: area under the curve. X-ray confirmed CAP is defined as presence of at least 2 clinical criteria and signs of an infiltrate on chest X-ray.
(TIFF)

S1 Table Factors associated with obtaining blood cultures. (DOCX)

## References

[1] Capelastegui A, Espana PP, Bilbao A, Gamazo J, Medel F, Salgado J, et al Study of community-acquired pneumonia: incidence, patterns of care, and outcomes in primary and hospital care. J Infect. Pneumology Service Hospital Galdakao, 48960 Galdakao, Bizkaia, Spain. alberto.capelasteguisaiz@osakidetza.net; 2010;61: 364–371. 10.1016/j.jinf.2010.07.015 20692290

[2] Colice GL, Morley MA, Asche C, Birnbaum HG. Treatment costs of community-acquired pneumonia in an employed population. Chest. Pulmonary, Critical Care and Respiratory Services, Washington Hospital Center, Washington, DC, USA. Gene.Colice@Medstar.net; 2004;125: 2140–2145. 15189934 10.1378/chest.125.6.2140

[3] Lim WS, Baudouin S V, George RC, Hill AT, Jamieson C, Le I J, et al BTS guidelines for the management of community acquired pneumonia in adults: update 2009. Thorax. Respiratory Medicine, Nottingham University Hospitals, David Evans Building, Hucknall Road, Nottingham NG5 1PB, UK. weishen.lim@nuh.nhs.uk; 2009;64 Suppl 3: iii1–55. 10.1136/thx.2009.121434 19783532

[4] Mandell LA, Wunderink RG, Anzueto A, Bartlett JG, Campbell GD, Dean NC, et al Infectious Diseases Society of America/American Thoracic Society consensus guidelines on the management of community-acquired pneumonia in adults. Clin Infect Dis. McMaster University Medical School, Hamilton, Ontario, Canada. lmandell@mcmaster.ca; 2007;44 Suppl 2: S27–S72. 17278083 10.1086/511159 PMC7107997

[5] Wiersinga WJ, Bonten MJ, Boersma WG, Jonkers RE, Aleva RM, Kullberg BJ, et al SWAB/NVALT (Dutch Working Party on Antibiotic Policy and Dutch Association of Chest Physicians) guidelines on the management of community-acquired pneumonia in adults. Neth J Med. Department of Internal Medicine, University of Amsterdam, Amsterdam, the Netherlands. w.j.wiersinga@amc.uva.nl; 2012;70: 90–101. 22418758

[6] Paolucci M, Landini MP, Sambri V. Conventional and molecular techniques for the early diagnosis of bacteraemia. Int J Antimicrob Agents. Department of Hematology and Oncology L e A Seragnoli, University of Bologna, St. Orsola University Hospital, Bologna, Italy; 2010;36 Suppl 2: S6–16. 10.1016/j.ijantimicag.2010.11.010 21129933

[7] Corbo J, Friedman B, Bijur P, Gallagher EJ. Limited usefulness of initial blood cultures in community acquired pneumonia. Emerg Med J. Department of Emergency Medicine, Albert Einstein College of Medicine, Bronx, New York, USA. jillcorbo@AOL.com; 2004;21: 446–448. 15208227 PMC1726373

[8] Skerrett SJ. Diagnostic testing for community-acquired pneumonia. Clin Chest Med. 1999;20: 531–48. 10516902 10.1016/s0272-5231(05)70234-3

[9] Falguera M, Trujillano J, Caro S, Menéndez R, Carratalà J, Ruiz-González A, et al A prediction rule for estimating the risk of bacteremia in patients with community-acquired pneumonia. Clin Infect Dis. 2009;49: 409–16. 19555286 10.1086/600291

[10] Marrie TJ, Durant H, Yates L. Community-acquired pneumonia requiring hospitalization: 5-year prospective study. Rev Infect Dis. 1989;11: 586–99. 2772465 10.1093/clinids/11.4.586

[11] Campbell SG, Marrie TJ, Anstey R, Dickinson G, Ackroyd-Stolarz S. The contribution of blood cultures to the clinical management of adult patients admitted to the hospital with community-acquired pneumonia: a prospective observational study. Chest. 2003;123: 1142–50. 12684305 10.1378/chest.123.4.1142

[12] LeBude B, Diemer G. Routine blood cultures for the febrile inpatient: a teachable moment. JAMA Intern Med. 2014;174: 1546–7. 10.1001/jamainternmed.2014.3687 25110969

[13] Coburn B, Morris AM, Tomlinson G, Detsky AS. Does this adult patient with suspected bacteremia require blood cultures? JAMA. Department of Medicine, Division of Infectious Diseases, University of Toronto, Toronto, Ontario, Canada; 2012;308: 502–511. 10.1001/jama.2012.8262 22851117

[14] Previsdomini M, Gini M, Cerutti B, Dolina M, Perren A. Predictors of positive blood cultures in critically ill patients: a retrospective evaluation. Croat Med J. Intensive Care Unit, Ospedale Regionale Bellinzona e Valli, Bellinzona, Switzerland. marco.previsdomini@eoc.ch; 2012;53: 30–39. 22351576 10.3325/cmj.2012.53.30 PMC3284177

[15] Shapiro NI, Wolfe RE, Wright SB, Moore R, Bates DW. Who needs a blood culture? A prospectively derived and validated prediction rule. J Emerg Med. Department of Emergency Medicine, Beth Israel Deaconess Medical Center, Boston, Massachusetts 02215, USA; 2008;35: 255–264. 10.1016/j.jemermed.2008.04.001 18486413

[16] Lee C-C, Wu C-J, Chi C-H, Lee N-Y, Chen P-L, Lee H-C, et al Prediction of community-onset bacteremia among febrile adults visiting an emergency department: rigor matters. Diagn Microbiol Infect Dis. 2012;73: 168–73. 10.1016/j.diagmicrobio.2012.02.009 22463870

[17] Metersky ML, Ma A, Bratzler DW, Houck PM. Predicting bacteremia in patients with community-acquired pneumonia. Am J Respir Crit Care Med. 2004;169: 342–7. 10.1164/rccm.200309-1248OC 14630621

[18] Van Werkhoven CH, Postma DF, Oosterheert JJ, Bonten MJM. Antibiotic treatment of moderate-severe community-acquired pneumonia: design and rationale of a multicentre cluster-randomised cross-over trial. Neth J Med. 2014;72: 170–8. 24846935

[19] Huijts SM, Pride MW, Vos JMI, Jansen KU, Webber C, Gruber W, et al Diagnostic accuracy of a serotype-specific antigen test in community-acquired pneumonia. Eur Respir J. 2013;42: 1283–90. 10.1183/09031936.00137412 23397295

[20] Muggeo VMR. Estimating regression models with unknown break-points. Stat Med. 2003;22: 3055–71. 10.1002/sim.1545 12973787

[21] Van Buuren S, Groothuis-Oudshoorn K. mice: Multivariate Imputation by Chained Equations in R. J Stat Softw. 2011;45: 1–67.

[22] R Core Team (2013). R: A language and environment for statistical computing. [Internet]. R Foundation for Statistical Computing, Vienna, Austria; 2013. Available: http://www.r-project.org/.

[23] Lim WS, van der Eerden MM, Laing R, Boersma WG, Karalus N, Town GI, et al Defining community acquired pneumonia severity on presentation to hospital: an international derivation and validation study. Thorax. Respiratory Infection Research Group, Respiratory Medicine, Nottingham City Hospital, Nottingham NG5 1PB, UK; 2003;58: 377–382. 12728155 10.1136/thorax.58.5.377 PMC1746657

[24] Müller B, Harbarth S, Stolz D, Bingisser R, Mueller C, Leuppi J, et al Diagnostic and prognostic accuracy of clinical and laboratory parameters in community-acquired pneumonia. BMC Infect Dis. 2007;7: 10 10.1186/1471-2334-7-10 17335562 PMC1821031

