# Supplementary figures and images for: Predictors of Bacteraemia in Patients with Suspected Community-Acquired Pneumonia
Source: PLoS One. 2015 Nov 24;10(11):e0143817. doi: 10.1371/journal.pone.0143817 (PMC4658054; doi:10.1371/journal.pone.0143817)

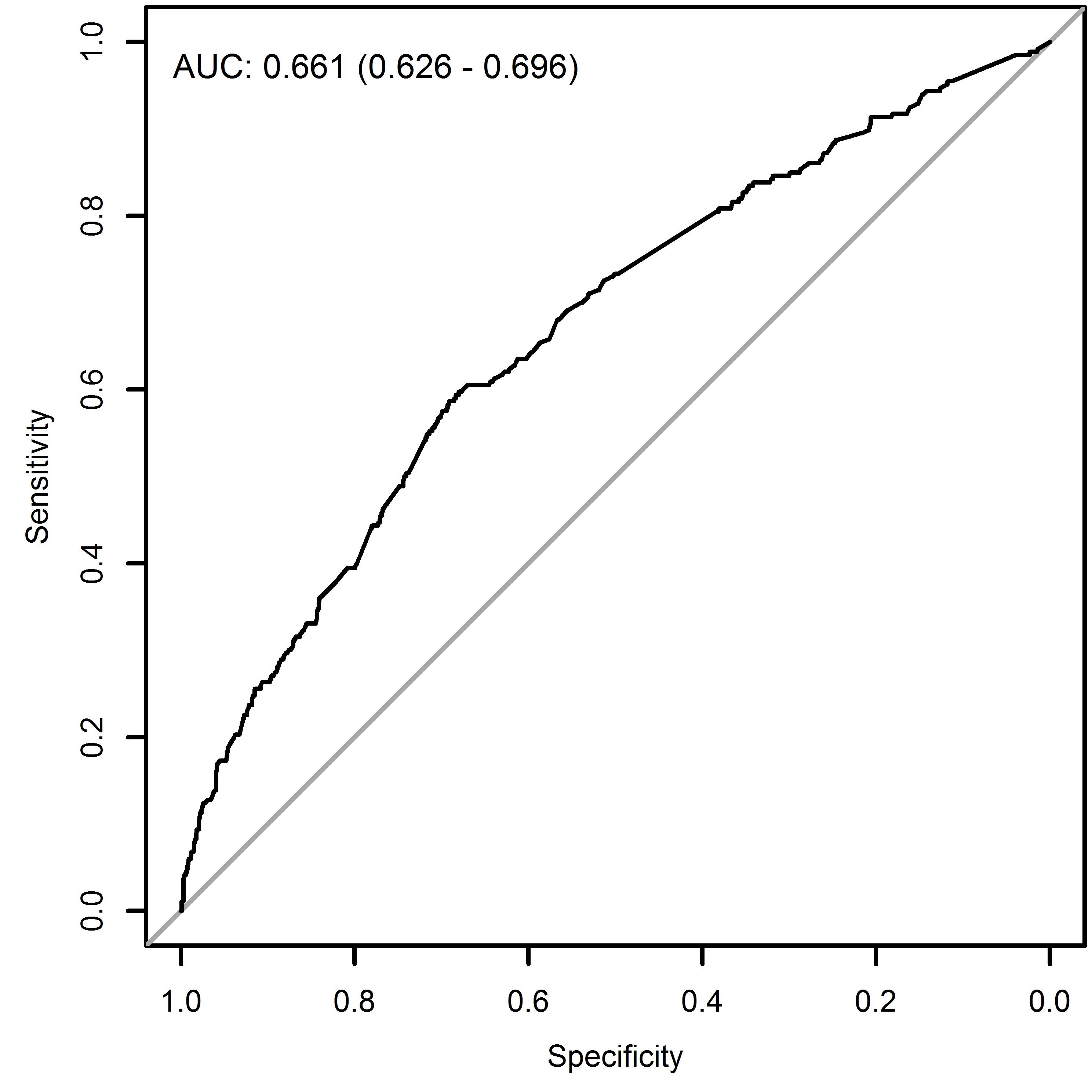

Supplement: S1 Fig — Abbreviations: ROC: receiver operating characteristic; AUC: area under the curve. (TIFF) [file pone.0143817.s002.tiff]

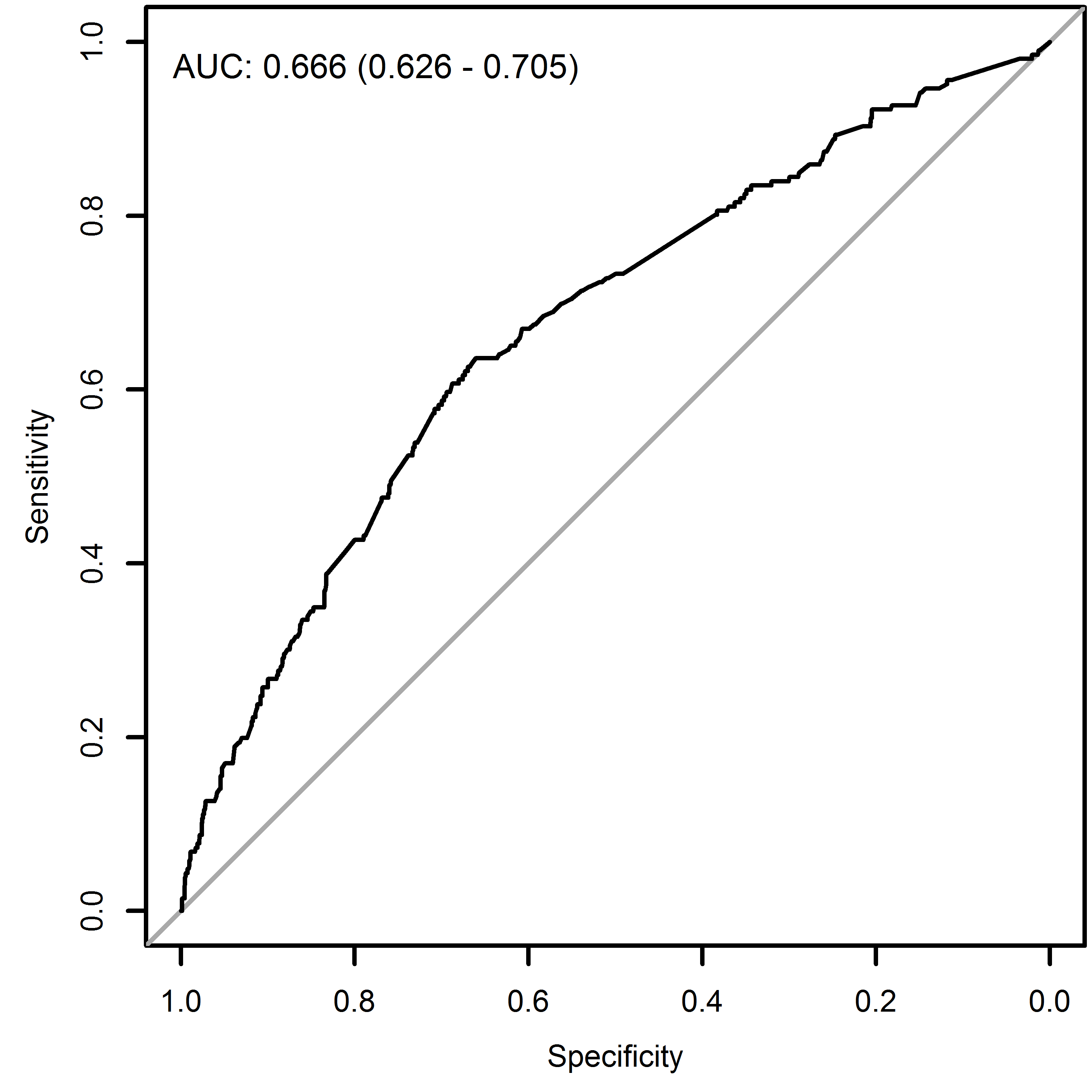

Supplement: S2 Fig — Abbreviations: ROC: receiver operating characteristic; AUC: area under the curve. X-ray confirmed CAP is defined as presence of at least 2 clinical criteria and signs of an infiltrate on chest X-ray. (TIFF) [file pone.0143817.s003.tiff]

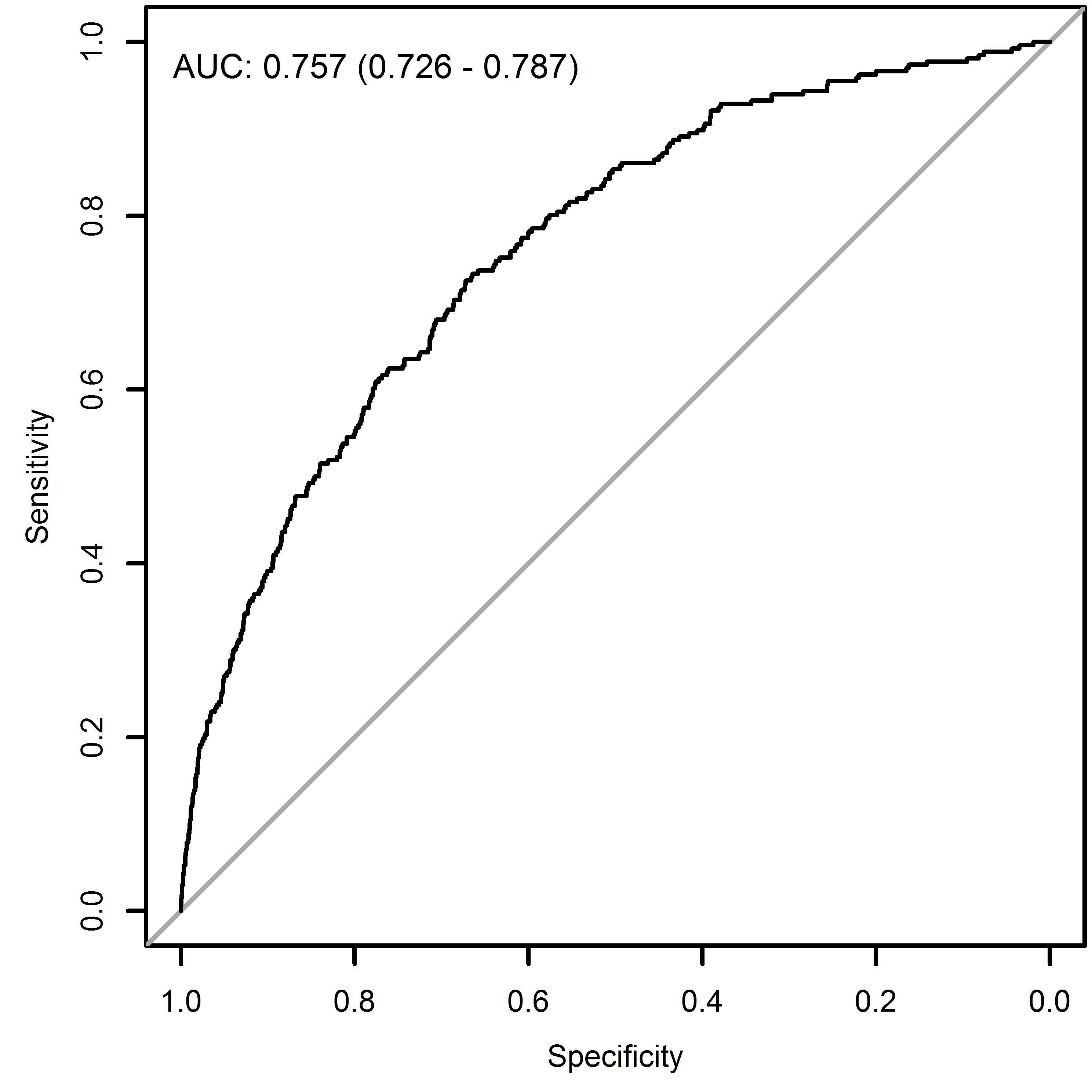

Supplement: S3 Fig — Abbreviations: ROC: receiver operating characteristic; AUC: area under the curve. (TIFF) [file pone.0143817.s004.tiff]

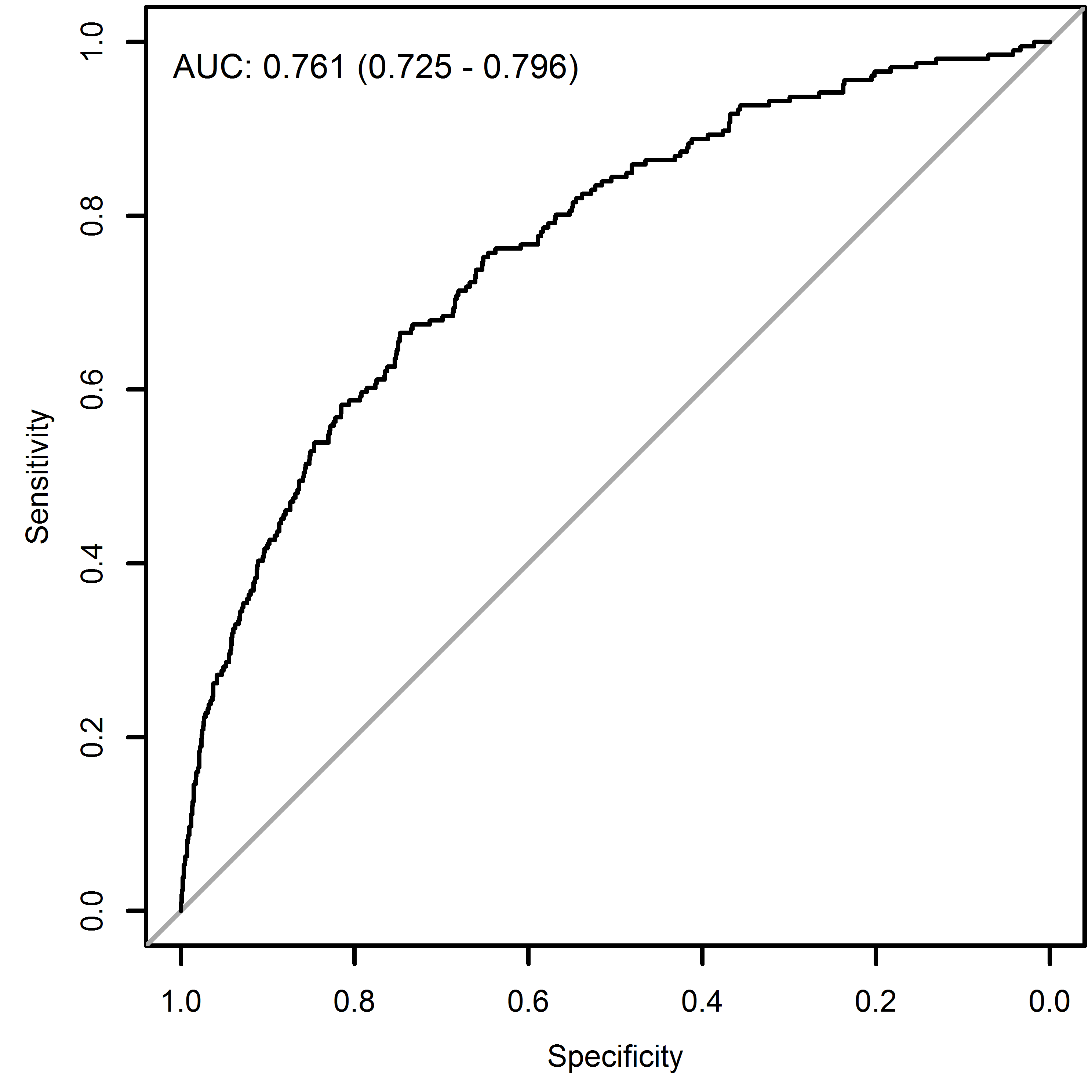

Supplement: S4 Fig — Abbreviations: ROC: receiver operating characteristic; AUC: area under the curve. X-ray confirmed CAP is defined as presence of at least 2 clinical criteria and signs of an infiltrate on chest X-ray. (TIFF) [file pone.0143817.s005.tiff]
