# Supplementary material for: Predictors of Bacteraemia in Patients with Suspected Community-Acquired Pneumonia
Source: PLoS One. 2015 Nov 24;10(11):e0143817. doi: 10.1371/journal.pone.0143817 (PMC4658054; doi:10.1371/journal.pone.0143817)
Supplement: S1 Table — (DOCX) [file pone.0143817.s006.docx]

**S1 Table. Factors associated with obtaining blood cultures**

| **Variable** | **OR** | **95% CI** | **Contribution to AUC #** |
| --- | --- | --- | --- |
| Age * | 0.990 | (0.984 to 0.997) | 0.2% |
| Chills | 1.661 | (1.252 to 2.204) | 0.2% |
| Confusion | 1.386 | (0.992 to 1.936) | 0.0% |
| Tachypnea | 1.296 | (0.955 to 1.758) | 0.0% |
| Temperature (values < 37 degrees Celcius) * | 0.670 | (0.509 to 0.882) | 14.7% |
| Temperature (values > 37 degrees Celcius) * | 4.131 | (3.571 to 4.779) | † |
| C-reactive protein (mg/L) $ | 1.010 | (1.002 to 1.018) | 0.1% |
| Leukocyte count (values < 10*10^9/L) * | 0.914 | (0.859 to 0.972) | 0.4% |
| Leukocyte count (values > 10*10^9/L) *^ | 1.038 | (1.020 to 1.055) | † |
| Thrombocyte count (values < 500*10^9/L) * | 0.998 | (0.997 to 0.999) | 0.4% |
| Thrombocyte count (values > 500*10^9/L) * | 1.001 | (0.998 to 1.004) | † |
| Blood glucose level (mmol/L)* | 1.029 | (0.998 to 1.061) | 0.1% |
| OR: odds ratio. CI: confidence interval. AUC: Area Under the Received Operator Characteristic Curve. # Contribution to the AUC was calculated by comparing the full model to the model without each variable. * OR per one unit increase. $ OR per 10 units increase. ^ Right-truncated at 40*10^9/L. † For these variables piecewise linear transformation was used, yielding two effect estimates, but they are considered one variable when calculating contribution to the AUC. | | | |
